# Classical swine fever virus nonstructural protein p7 modulates infectious virus production

**DOI:** 10.1038/s41598-017-13352-w

**Published:** 2017-10-11

**Authors:** Cheng Zhao, Xiaofang Shen, Rui Wu, Ling Li, Zishu Pan

**Affiliations:** State Key Laboratory of Virology, College of Life Sciences, Wuhan University, Wuhan, 430072 China

## Abstract

The classical swine fever virus (CSFV) nonstructural protein p7 is crucial for virus production, yet precisely how the p7 modulates this process is unclear. In this study, we first identified the interactions of p7 with E2 and NS2. The key binding regions of both p7 and NS2 mapped to the first transmembrane (TM1) domain of two proteins. Three amino acid substitutions in the TM1 region of p7 (p7^TDI18/19/20AAA^, p7^EVV21/22/23AAA^ and p7^YFY25/26/30AAA^) impaired infectious virus production and reduced the interaction of p7 with the NS2 protein. The E2p7 processing and mature p7, but not the E2p7 precursor, are essential for infectious virus production. Bicistronic mutants (pSM/E2/IRES) with single substitutions at residues 1 to 9 of p7 exhibited a significantly increased infectious CSFV titer compared to their counterparts in the context of pSM. Viral genomic RNA copies of the mutants exhibited similar levels compared with the wt CSFV. Our results demonstrated that CSFV p7 and its precursor E2p7 modulate viral protein interactions and infectious virus production without influencing viral RNA replication.

## Introduction

Classical swine fever virus (CSFV) is the causative agent of classical swine fever (CSF), a highly contagious viral disease of swine. CSFV, bovine viral diarrhea virus (BVDV) and border disease virus (BDV) are classified as members of the genus *Pestivirus* within the family *Flaviviridae* ^1^. CSFV is a single-stranded, positive-sense RNA virus with a genome of approximately 12.3 kb. The genome contains a single large open reading frame (ORF) encoding a polyprotein of approximately 3898 amino acids (aa) that is co- and post-translationally processed into at least 12 mature proteins^2,3^. Core, E^rns^, E1 and E2 are the pestivirus structural proteins^3,4^ and the nonstructural proteins (NS) NS3 to NS5B are necessary for viral RNA replication^5–7^.

Two additional nonstructural proteins, p7 and NS2, are located between the E2 and NS3 proteins of *Pestivirus*. The protein p7 is a small hydrophobic polypeptide with an apparent molecular mass of 6–7 kDa^8^. p7 has a homologue in *Hepcivirus* genus, but not in the *Flavivirus* genus^9,10^. For hepatitis C virus (HCV), p7 is involved in the release of infectious virions and virulence^8,11–14^. HCV p7 is essential for virus production but not for viral RNA replication and plays a role at an early stage of viral morphogenesis^14–17^. Upon HCV genome translation, p7 processing is mediated by host-encoded proteases^18,19^. However, cleavage at E2p7, which is performed by a unique mammalian signal peptidase, is incomplete, leading to the presence of both E2 and p7 mature forms, as well as some E2p7^9,20,21^. The efficiency of E2p7 processing modulates the production of infectious HCV^16,17,22^. NS2 is a transmembrane protein that contains an auto-protease responsible for cis-cleavage at the NS2/3 junction^23,24^. HCV p7 participates in virion assembly by interacting with NS2 and E2^25,26^. In addition, p7 regulates NS2 and Core subcellular localization independent of ion channel activity^22,27–29^. The nature of the p7 and E2p7 proteins and the peculiar processing of the E2p7NS2 region are well conserved among pestiviruses and HCV, which indicates a common function for these proteins^8^. CSFV p7 is a polytopic membrane protein having its N and C termini oriented toward the endoplasmic reticulum (ER) lumen^13,30^. The C-terminal transmembrane helix of p7 plays an important role in pore formation activity^11,13,30^. CSFV p7 is also involved in the virulence *in vivo* ^11,31^.

Here we investigated the effect of p7-mediated viral protein interactions and E2p7 processing on infectious CSFV production. Our results demonstrated that p7 modulates infectious CSFV production by either interacting with NS2 and E2 or regulating E2p7 cleavage.

## Results

### Identification of physical interactions between CSFV E2, p7 and NS2

**Figure 1:**
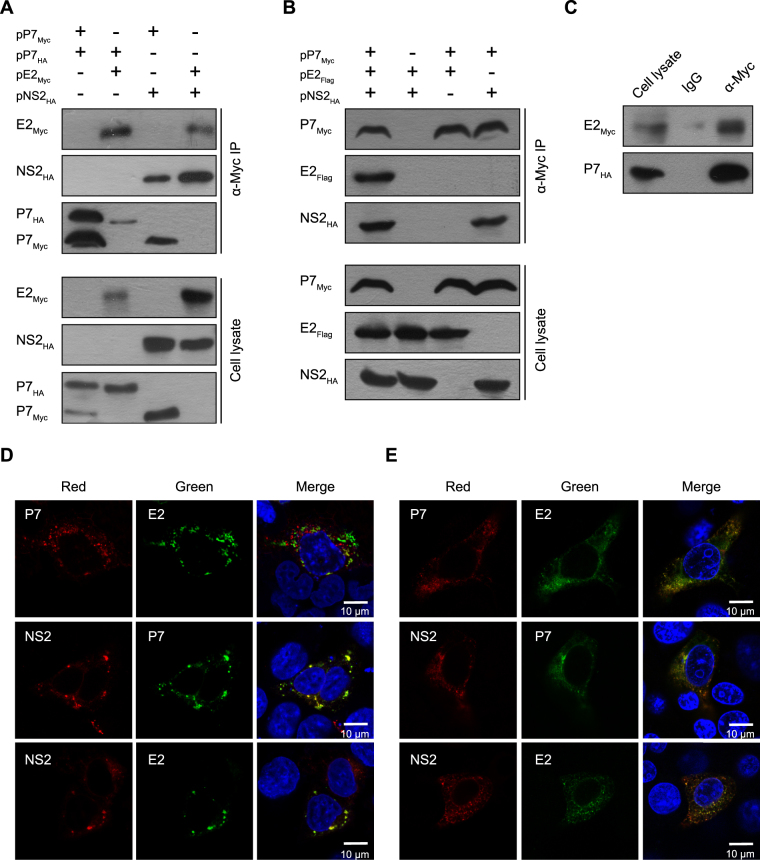
The interaction between p7 and E2 and/or NS2 *in vitro*. (**A**) p7 co-immunoprecipitates with E2, NS2 or/and p7 in 293 T cells. At 24 hours post-transfection (hpt), the transfected cells were lysed and immunoprecipitated with an anti-Myc antibody. The immunoprecipitates were separated by SDS-PAGE and analyzed by western blotting using an anti-HA antibody. (**B**) p7 forms a complex with E2 and NS2 in 293 T cells. (**C**) The interaction of p7 with E2 in SK6 cells. (**D**) The co-localization of p7 with E2 or NS2 in 293 T cells. The 293 T cells transfected with the indicated plasmids were immunostained with an anti-Myc or anti-HA antibody, respectively. The nuclei were stained with DAPI. Scale bar, 10 μm. (**E**) The co-localization of p7 with E2 or NS2 in PK15 cells.

The structural prediction indicated that CSFV p7 is a small hydrophobic protein that contains two hydrophobic regions connected by a polar segment^13^. Both p7 and NS2 proteins are essential for the production of infectious virions, but dispensable for RNA replication^6,11,32^. To investigate the effect of viral protein interactions on infectious virus production, we constructed antigenically tagged E2, p7 and NS2 eukaryotic expression plasmids that were used in co-immunoprecipitation (Co-IP) assays. Interactions between p7 and NS2, E2 and NS2, or p7 and p7 were observed in 293 T cells, but p7 exhibited a weak interaction with E2 (Fig. 1A). When 293 T cells were co-transfected with two or three expression plasmids corresponding to E2, p7 and NS2, NS2 or E2 with NS2 co-immunoprecipitated with the p7 protein, but that E2 hardly co-immunoprecipitated with the p7 protein in the absence of NS2 (Fig. 1B). When SK6 cells were co-transfected with the E2 and p7 expression plasmids, p7 co-immunoprecipitated with the E2 protein (Fig. 1C), suggesting that the physical interaction between CSFV E2 and p7 is cell type specific.

Next, we assessed the subcellular co-localization of p7 with E2 or NS2. When the antigenically tagged E2, p7 and NS2 plasmids were co-transfected into 293 T or PK15 cells, all three proteins were located in the cytoplasm and the subcellular co-localization of p7 and NS2, or E2 and NS2 was observed in both cell lines (Fig. 1D). Interestingly, the co-localization of E2 and p7 was only observed in PK15 cells (Fig. 1D), which confirmed data from Co-IP assay.

### Mapping the interaction regions of p7 and NS2

**Figure 2:**
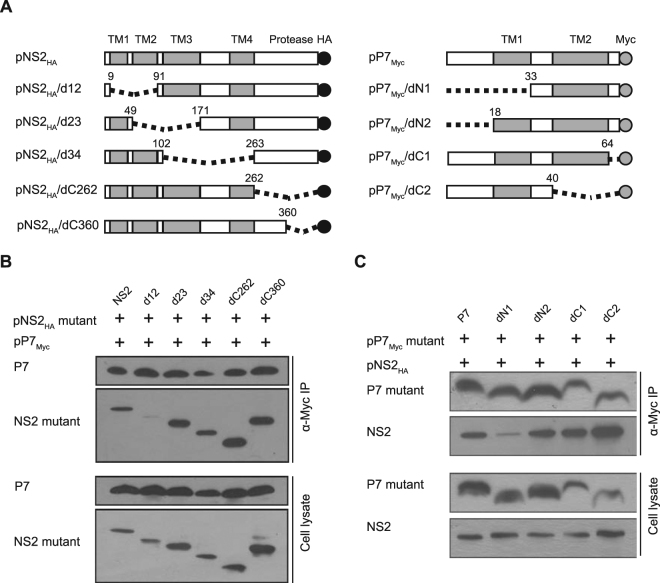
Mapping the interaction regions of p7 and NS2. (**A**) A schematic of the pP7_Myc_ and pNS2_HA_ expression constructs used in this study. Transmembrane regions, Myc-tag or HA-tag are depicted as gray or black, the number represents the position of amino acid residues. (**B**) The interaction of the mutated NS2 with p7 in 293 T cells. The cells transfected with indicated plasmids were lysed and immunoprecipitated with an anti-Myc antibody. (**C**) The interaction of the mutated p7 with NS2 in 293 T cells.

To map the interaction regions of p7 and NS2, a series of truncated mutants of p7 and NS2 were constructed (Fig. 2A). Based on the topology model of NS2^23^, the different transmembrane domains were deleted to generate NS2/d12 (deletion of TM1 and TM2 domains), NS2/d23 (deletion of TM2 and TM3 domains), NS2/d34 (deletion of TM3 and TM4 domains) and the C-termini were truncated to generate NS2/dC262, or NS2/dC360. Similarly, different N-terminal or C-terminal deletions of p7 were made to generate p7/dN1 and p7/dN2, or p7/dC1 and p7/dC2, respectively. Co-IP data showed that although none of the NS2 mutations resulted in a complete loss of p7-binding, the truncated NS2/d12 mutant exhibited a significantly decreased interaction with p7 compared to the wild-type (wt) NS2 (Fig. 2B). Similarly, the truncated p7 mutant containing the N-terminal TM1 domain deletion, p7/dN1, exhibited a significantly decreased interaction with NS2 compared with wt p7 or other truncated p7 mutants (Fig. 2C), suggesting that interaction regions of p7 and NS2 are located in the TM1 domains of both p7 and NS2.

**Figure 3:**
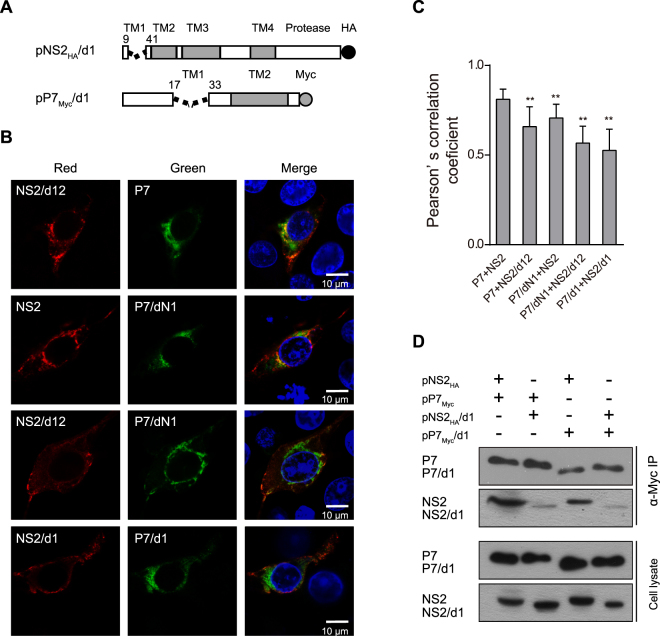
The interaction between the p7/d1 and NS2/d1 mutants. (**A**) A schematic of the pP7_Myc_/d1 and pNS2_HA_/d1 expression constructs used in this study. (**B**) The subcellular co-localization of p7/d1 with NS2/d1 in PK15 cells. The PK15 cells transfected with indicated plasmids were immunostained with both anti-Myc antibody (green) and anti-HA antibody (red) at 24 hpt. The nuclei were stained with DAPI. Scale bar, 10 μm. (**C**) The degree of co-localization was quantified by determining Pearson’s correlation coefficients. For each sample 30 cells were quantified using Image Pro Plus software. The error bars indicate standard deviations. (**D**) The interaction of p7/d1 with the NS2/d1 mutant. *P < 0.05; **P < 0.01; ns, not significant.

To further confirm the key binding region, the TM1 regions of both NS2 and p7 were deleted to generate the truncated mutants NS2/d1 and p7/d1, respectively (Fig. 3A). Co-localization analysis showed that p7/d1 and NS2/d1 exhibited significantly decreased subcellular co-localization compared with other mutants (Fig. 3B and C). Co-IP data showed that the interaction between p7/d1 and NS2/d1 was barely observable (Fig. 3D). Taken together, these data suggested that both the p7 TM1 region (aa 18–32) and the NS2 TM1 region (aa 10–40) are the major domains mediating the p7-NS2 interaction.

### The effect of amino acid mutation within the p7 TM1 region on infectious virus production

**Figure 4:**
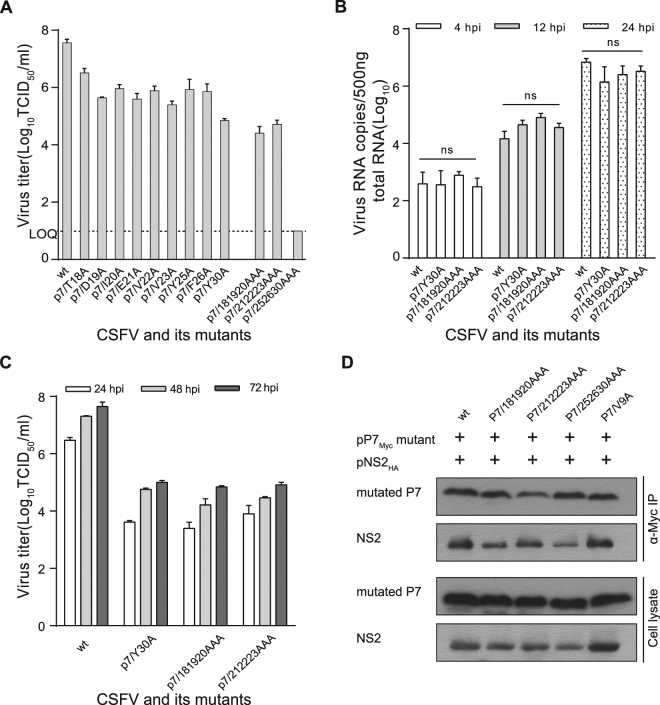
The effect of amino acid mutations in the p7 TM1 domain on infectious virus production. (**A**) PK15 cells were infected with the p7 TM1 region mutant virus at an MOI of 0.01 and virus titers were quantified by a limiting dilution assay. The viral titers in supernatants were determined by titration at 72 hpi and were expressed as TCID_50_/ml. LOQ: the limit of quantification. (**B**) The quantitative detection of viral RNAs. PK-15 cell monolayers were infected with the virus at an MOI of 0.001 and total RNA was extracted from the cells at 4, 12 and 24 hpi and 5,000 ng of total RNA was reverse transcribed (RT). Next, 2 μl of viral cDNA (500 ng of total RNA) was analyzed by the quantitative SYBR RT-PCR assay. (**C**) Virus titers were determined at the indicated time points. The error bars indicate standard deviations. (**D**) The interaction between the p7 TM1 domain mutant and NS2. 293 T cells were transfected with the indicated plasmids followed by Co-IP assays using an anti-Myc antibody.

To investigate the role of the p7 TM1 domain in infectious virus production, we generated a panel of amino acid substitution mutants based on the conservation, charge, polarity and hydrophobicity of amino acids and previous reports of lethal p7 mutants^11^. The mutated cDNA clones containing a single amino acid substitution (p7^T18A^, p7^D19A^, p7^I20A^, p7^E21A^, p7^V22A^, p7^V23A^, p7^Y25A^, p7^F26A^ or p7^Y30A^) or three amino acids substitutions (p7^TDI18/19/20AAA^, p7^EVV21/22/23AAA^ or p7^YFY25/26/30AAA^) were constructed using the infectious cDNA clone pSM as a template, respectively. Rescued CSFVs were detected by immunofluorescence (IF) assays with an anti-NS3 antibody^33^. The PK15 cells transfected with these transcripts were positive for viral antigen staining (supplementary Fig. 2). The infectious virus in the supernatant of the transfected PK15 cells was titrated on PK15 cells at 72 hours post-infection (hpi). Data showed that all mutants exhibited significantly decreased virus titers compared with the wt CSFV, especially the mutants p7^Y30A^, p7^TDI18/19/20AAA^ or p7^EVV21/22/23AAA^, while the mutant p7^YFY25/26/30AAA^ blocked infectious virus production almost completely (Fig. 4A). The p7 amino acid sequences of mutated viruses remained unchanged after 5 serial passages in PK15 cells (data not shown). The viral RNAs in PK15 cells infected with the mutants p7^Y30A^, p7^TDI18/19/20AAA^, or p7^EVV21/22/23AAA^ were quantified by qRT-PCR and the virus titers were determined using IF assays at the indicated time points. Data showed that the mutants exhibited similar viral RNA levels and significantly decreased virus titers compared with the wt CSFV (Fig. 4B and C). However, an attempt to detect viral RNA copies in PK15 cells transfected with the mutant p7^YFY25/26/30AAA^ failed due to the lethal phenotype.

To understand the relationship between the p7-NS2 interaction and infectious virus production, we constructed the mutated eukaryotic expression plasmids pP7^TDI18/19/20AAA^, pP7^EVV21/22/23AAA^ and pP7^YFY25/26/30AAA^ using pP7_Myc_ as a template. The N-terminal mutant p7^V9A^ was used for comparison. Co-IP data showed that the mutated p7 proteins, p7^TDI18/19/20AAA^, p7^EVV21/22/23AAA^ and p7^YFY25/26/30AAA^ exhibited notably decreased interactions with NS2, while interaction between p7^V9A^ and NS2 remained unaltered (Fig. 4C), suggesting that the amino acids located in the p7 TM1 region regulate infectious virus production by mediating the interaction with NS2.

### E2p7 facilitates the interaction between NS2 and E2 and infectious virus production

**Figure 5:**
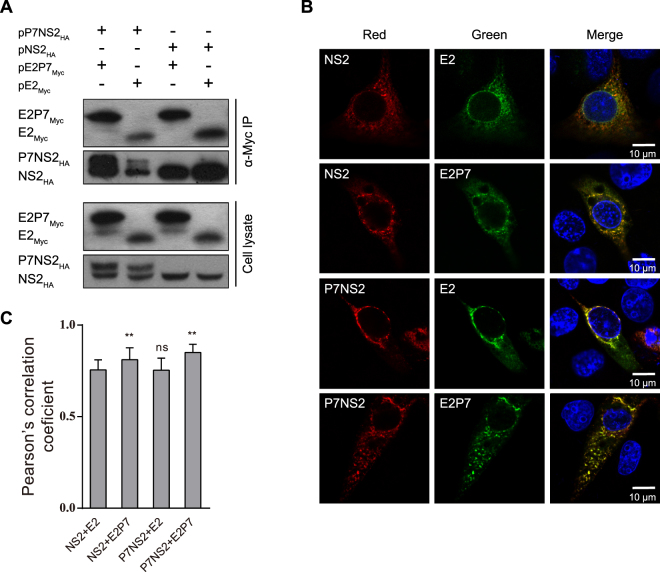
The interaction between E2p7 and p7NS2. (**A**) The interaction between E2p7 and p7NS2 by a Co-IP assay. 293 T cells transfected with indicated plasmids were lysed and immunoprecipitated with an anti-Myc antibody. (**B**) The subcellular co-localization between E2p7 and NS2 in PK15 cells. PK15 cells transfected with indicated plasmids were immunostained with both an anti-Myc antibody (green) and an anti-HA antibody (red) at 24 hpt. The nuclei were stained with DAPI. Scale bar, 10 μm. (**C**) The degree of co-localization was quantified by determining Pearson’s correlation coefficients. For each sample 30 cells were quantified using Image Pro Plus software. Data are represented as the mean values (±SD) from three independent experiments. *P < 0.05; **P < 0.01; ns, not significant.

For *Flaviridae*, the E2p7 precursor exists during the virus life cycle^8,15,22,34^. To explore the effect of E2p7 on virus protein interaction, we constructed the antigenically tagged p7NS2 and E2p7 eukaryotic expression plasmids. Co-IP data showed that the interaction between E2p7 and p7NS2 or E2p7 and NS2 was significantly increased compared with that observed between E2 and NS2. However, the interaction between E2 and p7NS2 exhibited obviously decreased compared with that observed between E2 and NS2 (Fig. 5A). Similarly, co-localization analysis showed that the subcellular co-localization of E2p7 and p7NS2 or E2p7 and NS2 was significantly enhanced compared with that observed between E2 and NS2. In contrast, the subcellular co-localization between E2 and p7NS2 remained unchanged compared with that observed between E2 and NS2 (Fig. 5B and C). These results indicated that E2p7 precursor facilitates the interaction of NS2 with E2 or E2p7 in PK15 cells.

**Figure 6:**
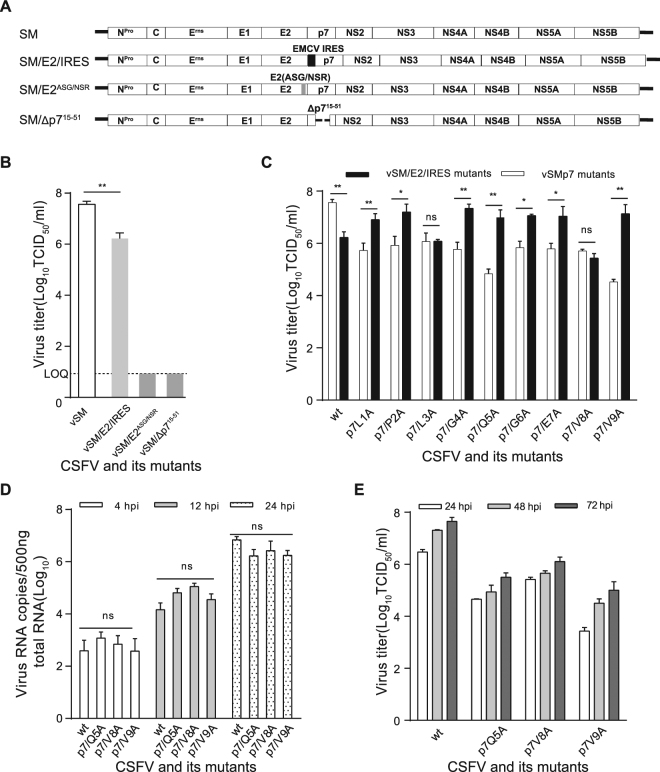
The effect of single amino acid mutations in the p7 N-terminus on infectious CSFV production and genomic RNA replication. (**A**) Construction of the modified CSFV cDNA clones, pSM/E2/IRES, pSM/E2^ASG/NSR^ or pSM/Δp7^15–51^. (**B**) PK15 cells were infected with indicated mutant viruses at an MOI of 0.01 and the viral titers in supernatants were determined at 72 hpi and expressed as TCID_50_/ml. LOQ: limit of quantification. (**C**) Infectious viruses in supernatants of PK15 cells infected with the p7 aa 1–9 mutant viruses were quantified by a limiting dilution assay at 72 hpi. The viral titers were expressed as TCID_50_/ml. (**D**) The quantitative detection of viral RNAs by qRT-PCR. Data represent the mean values (±SD) from three independent experiments. (**E**) Virus titers were determined at the indicated time points. The error bars indicate standard deviations. **P < 0.01; ns, not significant.

To investigate the role of E2p7 in infectious virus production, an IRES element was inserted between the E2 and p7 genes to generate a bicistronic cDNA clone, pSM/E2/IRES (Fig. 6A). The infectious CSFV was rescued from PK15 cells transfected with *in vitro*-transcribed RNA corresponding to pSM/E2/IRES. However, this modification resulted in a significantly decreased virus titer compared to the wt CSFV (Fig. 6B). When the E2p7 processing site (E2/NSR) was inactivated or the p7 15–51 aa region was deleted, no infectious CSFV was rescued from the mutated cDNA clones pSM/E2^ASG/NSR^ or pSM/Δp7^15–51^ (Fig. 6A and B). These results indicated that the release of E2 and p7 from E2p7 is essential for infectious CSFV production and that the presence of the E2p7 precursor significantly increases the virus titer.

Previous studies showed that mutation of p7 modulates the cleavage efficiency at the E2p7 junction^17,35^. To assess the role of amino acids located in the p7 N-terminus in E2p7 processing efficiency and infectious CSFV production, we screened the amino acid residues 1 to 9 adjacent to the cleavage junction by alanine mutagenesis in the context of the pSM or pSM/E2/IRES cDNA clones, respectively. The infectious CSFV was rescued from PK15 cells transfected with mutated genomic RNA and all of mutant viruses exhibited significantly decreased virus titers compared with the wt CSFV (Fig. 6C), whereas most mutants in the context of the pSM/E2/IRES clones yielded higher virus titers than their pSM counterparts. The p7^L1A^, p7^P2A^, p7^G4A^, p7^Q5A^, p7^G6A^, p7^E7A^, p7^V9A^ mutants in the context of pSM/E2/IRES exhibited significantly increased virus titers compared with those in the context of pSM. For the p7^L3A^ and p7^V8A^ mutants, similar infectious virus production was observed in the context of the pSM/E2/IRES and pSM clones (Fig. 6C). Viral RNAs of mutants were sequenced after 5 serial passages in PK15 cells and no reversion was observed in p7. The genomic RNAs of representative mutants (p7^Q5A^, p7^V8A^ and p7^V9A^) in the context of pSM were assessed by qRT-PCR. Data showed that viral genomic RNA copies of the mutants exhibited similar levels compared with the wt CSFV (Fig. 6D). Taken together, these results suggested that the E2p7 precursor facilitates infectious virus production and that the amino acids located in the N-terminus of p7 play a role in virus production, primarily by modulating E2p7 processing and protein interaction. Therefore, the modulation of p7 and E2p7 for infectious virus production occurred during virion maturation, independent of viral RNA replication.

## Discussion

In *Flaviviridae*, the p7 protein, as a multi-functional viroporin, is encoded only by *Pestivirus* and HCV^11–13,15,36,37^. The HCV p7 protein is necessary for the replication of infectious virus in animals and the secretion of infectious virions in cultured cells^14,16,38–40^. CSFV p7 is an integral membrane protein involved in virulence and its pore-forming activity resides in the C-terminal transmembrane helix^11,13^.

There is increasing genetic and biochemical evidence for the cooperation between p7 and NS2 during virus assembly. In HCV, the interactions of NS2 with E1, p7 and NS3 synergistically modulate virus assembly^41^. The p7 and NS2 proteins are key determinants governing the subcellular localization of the HCV core from lipid droplets (LDs) to the ER and are required for the initiation of the early steps of virus assembly^29^. CSFV NS2 has been recently reported to modulate the NS3/4A-kink interaction, which led to a less compact conformation required for virion morphogenesis^42^. In this study, we observed physical interactions of CSFV p7 with E2 and NS2 and the homo-oligomers of p7 protein in 293T cells for the first time (Fig. 1A). The key binding regions of p7 and NS2 mapped in the TM1 regions of both proteins. Interestingly, p7 clearly co-localized with E2 and NS2 in PK15 cells, but failed to co-localize with E2 in 293 T cells (Fig. 1D and E). Co-IP data in transfected SK6 cells confirmed this observation (Fig. 1C). These results suggested that the physical interaction between E2 and p7 is cell type specific and that CSFV p7 is involved in the formation of an E2, p7 and NS2 complex that is important for virus assembly. Similarly, the interactions of HCV proteins exist only in certain cell lines^26^ and HCV p7 mediates virion assembly by participating in the formation of an assembly complex^27,43–45^.

The cleavage at the *Flaviridae* E2p7 junction is incomplete, resulting in the formation of the proteins E2p7, p7 and E2^8,9,15,20,21,35^. For BVDV, p7 and E2p7 are both dispensable for viral RNA replication, but p7 is essential for infectious virus production^15^. The insertion of an IRES at the cleavage site of E2p7 led to significantly decreased infectious virus and no virus was recovered from the mutated cDNA clone that contained the deleted p7, pSM/Δp7^15–51^ or the inactivated E2p7 cleavage site, pSM/E2^ASG/NSR^ (Fig. 6B). These results indicated that both CSFV E2p7 processing and the p7 protein are essential for the generation of infectious virions. It has been confirmed that the effective release of E2 and p7 from the precursor E2p7 promotes HCV production by enhancing NS2-associated virus assembly complex formation near LDs^22^. Our data demonstrated that the E2p7 precursor facilitated the production of infectious virions, possibly by pulling mature E2 or p7 proteins that were released from the E2p7 precursor retained in the ER into the virion. In support of this hypothesis, E2p7 exhibited a higher binding capacity with NS2 and p7NS2 than E2 (Fig. 5A and B).

In the context of the pSM clone, all of single amino acid mutations in p7 residues 1–9, which are adjacent to the cleavage site, resulted in significantly reduced virus titers. Interestingly, most bicistronic mutants with the p7 single residue mutation exhibited an increased virus titer compared with their monocistronic counterparts and recovered to a similar virus titer as the wt vSM/E2/IRES (Fig. 6C), suggesting that a defect in E2p7 cleavage was at least partially responsible for the decrease of infectious virus production. In contrast, viral RNA replication remained unaffected. Our results demonstrated that regulation of p7 for virus production was not involved in viral RNA replication. A similar observation was made for HCV p7^17^.

The compatibilities between HCV p7 and the first NS2 transmembrane domain is required to induce Core-ER localization and the assembly of infectious viral particles^29^. When the representative mutants containing three amino acid substitutions in the CSFV p7 TM1 region were investigated, the mutants p7^TDI18/19/20AAA^ and p7^EVV21/22/23AAA^ exhibited significantly decreased viral titers compared with the single amino acid mutants, but no virus was rescued from the p7^YFY25/26/30AAA^ mutant (Fig. 4A). The interaction between the p7^TDI18/19/20AAA^, p7^EVV21/22/23AAA^ or p7^YFY25/26/30AAA^ mutant proteins with NS2 was notably reduced (Fig. 4C). Combined with the finding that the viral RNA replication levels of these mutants was comparable to that in wt CSFV (Fig. 4B), these data suggested that the amino acids located in the p7 TM1 region regulate infectious virus production by mediating the interaction with NS2. Furthermore, it is worth noting that even though no virus could be rescued from the p7^YFY25/26/30AAA^ mutant, PK15 cells transfected with *in vitro*-transcribed RNA corresponding to this mutant was positive for viral antigen staining. Because p7 is not involved in viral RNA replication^14–16,46–48^, we speculated that the weak interaction between p7^YFY25/26/30AAA^ and NS2 led to a defect in virus assembly and morphogenesis independent from genome RNA replication and translation. Similarly, mutation of HCV p7 impairs infectious virus production, while viral RNA replication is unaffected^17,22^. These results revealed an important regulatory function of the first transmembrane domains of p7 and NS2 for infectious virus assembly. Thus, we propose that the formation of an E2, p7 and NS2 complex at the ER membrane modulates infectious CSFV production (Supplementary Fig. 3).

In conclusion, the mature p7 and E2p7 cleavage are essential for the generation of infectious CSFV. CSFV p7 mediated protein interactions or E2p7 cleavage modulates virus production without influencing viral RNA replication. It is conceivable that CSFV p7 play a key role in viral life cycles by mediating E2, p7 and NS2 complex formation during virus assembly. The functional role of CSFV p7 in the context of infectious virions and the p7-mediated pathogenic mechanisms require further research.

## Materials and Methods

### Cell culture

Porcine kidney 15 (PK15), swine kidney cells (SK6) and human embryonic kidney cells (293 T) were obtained from the China Center for Type Culture Collection (CCTCC, Wuhan, China) and cultured in Dulbecco’s Modified Eagle Medium (DMEM) (Invitrogen, Carlsbad, CA,USA) supplemented with 10% fetal bovine serum (Gibco, Grand Island, NY, USA) and nonessential amino acids (NEAA) and contained penicillin (100 units/ml) and streptomycin (100 g/ml). Cells were grown at 37 °C in a humidified 5% CO_2_ atmosphere.

### Construction of plasmids

The CSFV E2, p7 or NS2 genes were amplified by polymerase chain reaction (PCR) using the infectious cDNA clone pSM as a template with the indicated primers (Supplementary Table 1). The PCR-amplified fragments were then cloned into different eukaryotic expression plasmids to generate pE2_Myc_, pE2_Flag_, pE2P7_Myc_, pP7_Myc_, pP7_HA_, pNS2_HA_ and pP7NS2_HA_. The truncated p7 or NS2 mutants were constructed by overlapping PCR.

The bicistronic cDNA clone (pSM/E2/IRES) that contained an internal ribosome entry site element (IRES), the cDNA clone (pSM/Δp7^15–51^) lacking the p7 amino acid residues 15–51 and the cDNA clone (pSM/E2^ASG/NSR^) containing E2 ASG to GSR mutations were all constructed by overlapping PCR using standard procedures^17,25,49^. Specific mutations in the p7 N-terminus were introduced by overlapping PCR using standard procedures and engineered into the infectious cDNA clone pSM. The corresponding p7 mutants were similarly constructed in the context of the bicistronic cDNA clone pSM/E2/IRES.

Details concerning the generation of constructs can be found in the supplementary material.

### *In vitro* transcription and transfection

*In vitro* transcription of CSFV genomic RNA was conducted as described previously^50,51^ using a T7 High Yield Transcription Kit (Life Technologies, Carlsbad, California, USA). Briefly, the *Mlu*I-linearized wild-type or mutated CSFV cDNA clone containing a T7 promoter was used as template for genomic RNA synthesis^51^. The RNA products were purified using a MEGAclear kit (Applied Biosystems, CA, USA). The integrity of the transcribed RNA was verified by agarose gel electrophoresis and the RNA concentration was measured by UV spectrophotometry (Thermo NanoDrop 2000c, USA).

DNA or RNA transfection was performed as described previously^50,51^. Briefly, 5 μl of Lipofectamine®2000 (Invitrogen) and 2.5 μg DNA or 1 μg RNA was diluted into 100 μl of Opti-MEM. The diluted DNA or RNA was added to each tube of diluted Lipofectamine, followed by incubation at room temperature for an additional 5 min. The 293 T or PK15 cell monolayers, grown on 6-well cell culture plates, were washed twice with Opti-MEM and incubated with the DNA or RNA transfection mixture for 6 h and then the culture medium containing the transfection mixture was replaced with fresh medium and incubated for 24 to 72 h for subsequent assays.

### Immunoprecipitation and western blotting

For the immunoprecipitation assays^43,52^, the transfected cells were harvested at 24 hours post-transfection (hpt) and washed with cold phosphate-buffered saline (PBS). The cells were lysed in lysis buffer (20 mM Tris-HCl, pH 7.4, containing 150 mM NaCl, 1% TritonX-100, 1 mM EDTA and protease inhibitors cocktail). Cell lysates were sonicated for 10 min and then incubated for 30 min at 4 °C, followed by centrifugation at 12,000 × g for 20 min. The supernatants were incubated with 2 µl anti-Myc antibody (A02060, Abbkine, Redlands, CA, USA) for 2 h at 4 °C. The immunocomplexes were precipitated with 25 μl of protein G (sc2002, Santa Cruz Biotechnology, Europe) for 2 h at 4 °C and washed three times with lysis buffer. The precipitated proteins were subjected to SDS-polyacrylamide gel electrophoresis (PAGE).

For western blotting^43^, proteins separated by SDS-PAGE were transferred to nitrocellulose membranes (Roche, Basel, Switzerland) and incubated with specific primary antibodies, followed by the addition of secondary antibody (HRP-conjugated goat anti-mouse light chain specific or mouse anti-rabbit IgG light chain specific) (Abbikine, Redlands, CA, USA). The signal was acquired using an enhanced chemiluminescence detection system (ECL, Bio-Rad, Hercules, CA, USA) as recommended by the manufacturer.

### Immunofluorescence and confocal microscopy

The transfected cells were fixed with 50% (v/v) methanol/acetone for 30 min at −20 °C at 24 to 48 hpt then were blocked in PBS with 3% BSA (Bovine serum albumin; Biosharp, Germany) and then incubated with the diluted primary antibody in 1% BSA for 2 h. After washing, the cells were incubated with the diluted secondary antibody (Alexa-488-Fluor-conjugated donkey anti-mouse or Alexa-594-Fluor-conjugated donkey anti-rabbit) (Invitrogen, Eugene, Oregon, USA) in 1% BSA for 1 h. Next, the cells were incubated with DAPI for 20 min to stain nuclei. Cells were examined with a LAS AF Lite 4.0 confocal laser scanning biological microscope (Leica, Solms, Germany).

### Virus titration

For virus titrations, serial 10-fold dilutions of clarified cell supernatants were inoculated in 100 μl into PK15 cells seeded at 1 × 10^4^ cells/well in 96-well plates (Nest, China). Cells were incubated at 37 °C for 1.5 h and then the virus dilution was replaced with DMEM supplemented with 2% fetal bovine serum. At 72 hpi, the cells were subjected to an immunofluorescence (IF) assay using an anti-NS3 antibody. Infectivity was determined using the method of Reed-Muench and expressed as TCID_50_/ml^53^.

### Quantitation of CSFV genomic RNA

CSFV genomic RNA was detected by a SYBR -Green I-based real-time RT-PCR assay^54^. Viral RNA replication was evaluated by quantifying viral RNA copies in infected cells by qRT-PCR^55^. PK15 cells were infected with the virus at an MOI of 0.001. Total RNA was extracted from the cells at 4, 12 and 24 hpi using TRIzol reagent (Invitrogen) and 5,000 ng of total RNA was reverse transcribed (RT) using random primers (N)_9_ (BioColors, Shanghai, China) and M-MLV reverse transcriptase (Promega, USA)^50^. Next, 2 μl of viral cDNA (500 ng of total RNA) was analyzed by qPCR using the primers: F98, 5′-CCATGCCCATAGTAGGACTAGCAAA-3′; and R202, 5′-TCACTGCGAACTACTACTGACGACTGT-3′. Reaction mixtures were incubated at 95 °C for 60 s, followed by 40 cycles of 95 °C for 15 s and 60 °C for 60 s. The results, represented as RNA copies, were recorded from three independent experiments and each experiment was performed in duplicate.

### Statistical analyses

All values are expressed as the means ± standard deviation (SD). Statistical significance for multiple comparisons was determined by one-way or two-way ANOVA, followed by Sidak’s multiple comparisons test in Prism (GraphPad Software, La Jolla, CA). Comparisons between two groups were performed with the unpaired Student’s t-test. A *P*-value of less than 0.05 was considered significant.

## Electronic supplementary material

Supplementary Information
