# Supplementary material for: Classical swine fever virus nonstructural protein p7 modulates infectious virus production
Source: Sci Rep. 2017 Oct 11;7:12995. doi: 10.1038/s41598-017-13352-w (PMC5636883; doi:10.1038/s41598-017-13352-w)
Supplement: Supplementary file 1 — Supplementary Information [file 41598_2017_13352_MOESM1_ESM.doc]

**Classical swine fever virus nonstructural protein p7 modulates infectious virus production**

Cheng Zhao, Xiaofang Shen, Rui Wu, Ling Li, Zishu Pan*

State Key Laboratory of Virology, College of Life Sciences, Wuhan University, Wuhan 430072, China

*To whom correspondence should be addressed.

Tel: 86-27-68752833

Fax: 86-27-68752833

Email: [zspan@whu.edu.cn](mailto:zspan@whu.edu.cn)

Mail to: State Key Laboratory of Virology, College of Life Sciences, Wuhan University, Wuhan 430072, China

**Construction of plasmids**

To investigate the interaction among E2, p7 and NS2, the eukaryotic expression plasmids with different antigenic tages, pE2_Myc_, pE2_Flag_, pE2p7_Myc_, pP7_Myc_, pP7_HA_, pNS2_HA_, and pP7NS2_HA_ were constructed, respectively. Briefly, E2 fragment was amplified by PCR from pSM template and the amplified product was cloned into pEE using restriction enzyme *Afl*II and *Mlu*I digestion to generate the pE2_Myc_ plasmid. Similarly, the expression plasmid, pE2_Flag_, pE2p7_Myc_, pP7_Myc_, pP7_HA_, pNS2_HA_ or pP7NS2_HA_, was constructed following primers and restriction enzymes description in Supplementary Table 1, respectively. All constructs were sequenced to confirm their identities.

**Supplementary Table 1** Primers for construction of antigenically tagged E2, p7, NS2 expression plasmids

| Primers | sequence (5'-3') | Constructs |
| --- | --- | --- |
| FE2_Myc_ | GCTCTTAAGGCCACCATGCGGCTAGCCTGCAAGGAAG (*Afl*II) | pE2_Myc_ |
| RE2_Myc_ | ATGCACGCGTACCAGCGGCGAGTTGTTCT (*Mlu*I) |
| FE2_Flag_ | GGCCACGCGTGCGGCCGCATGCGGCTAGCCTGCAAGGAAG (*Mlu*I) | pE2_Flag_ |
| RE2_Flag_ | AATCCTCGAGACCAGCGGCGAGTTGTT (*Xhol*I) |
| F_Myc_E2P7 | GCGGGATCCCGGCTAGCCTGCAAG (*BamH*I) | pE2p7_Myc_ |
| R_Myc_E2P7 | GTACCTCGAGTCAACCCTTGGCAACCCCG (*Xhol*I) |
| FP7_Myc_ | AGCTCTTAAGGCCACCATGCTACCATTGGGCCAG (*Afl*II) | pP7_Myc_ |
| RP7_Myc_ | ATGCACGCGTACCCTTGGCAACCCCGCTAAC (*Mlu*I) |
| FP7NS2_HA_ | TTCTAAGCTTGCCACCATGGGGCTACCATTGGGCCAG (*Hind*Ⅲ) | pP7_HA_ |
| RP7_HA_ | CATTCTAGATGCACGCGTACCCTTGGCAACCCCGCT (*Xba*I) |
| FNS2_HA_ | TTCTAAGCTTGCCACCATGGGGGGAAAGATAGATGGC (*Hind*Ⅲ) | pNS2_HA_ |
| RNS2_HA_ | CTAGAGTCGACTCTAAGCACCCAGCCAAG (*Sal*I) |
| FP7NS2_HA_ | TTCTAAGCTTGCCACCATGGGGCTACCATTGGGCCAG (*Hind*Ⅲ) | pP7NS2_HA_ |
| RNS2_HA_ | CTAGAGTCGACTCTAAGCACCCAGCCAAG (*Sal*I) |

The underlined sequences marked restriction enzyme sites

The truncated mutants of p7 or NS2 were constructed seperately for mapping the interaction region of p7 and NS2. For construction of p7 mutants, the fragment encoding amino acids 33-70, 18-70, 1-64 or 1-40 of p7 was amplified by PCR using primers FP7dN1/RP7_Myc_, or FP7dN2/RP7_Myc_, or FP7_Myc_/RP7dC1, or FP7_Myc_/RP7dC2, respectively. The PCR-amplified fragment was cloned into the pEF using restriction enzyme *Afl*II and *Mlu*I digestion to generate pP7_Myc_/dN1, pP7_Myc_/dN2, pP7_Myc_/dC1, pP7_Myc_/dC2, respectively (Supplementary Table 2). The p7 fragment containing deletion of p7 18-32 amino acids was amplified by overlapping PCR with specific primers, FP7d1B/RP7_Myc_, FP7d1A/RP7_Myc_, FP7_Myc_/RP7_Myc_ and then was cloned into pEF using the *Afl*II and *Mlu*I digestion to generate pP7_Myc_/d1 (Supplementary Table 2).

For construction of truncated NS2 mutants, the NS2 fragment containing deletion of NS2 10-90 amino acids was amplified by overlapping PCR with specific primers FpEF896/RNS2d12, FNS2d12/RNS2_HA_ and then cloned into the pKH3 plasmid using restriction enzyme *Spel*I and *Sal*I digestion to generate pNS2_HA_/d12. Similarly, pNS2_HA_/d1 containing deletion of NS2 10-40 amino acids and pNS2_HA_/d23 containing deletion of NS2 50-170 amino acids were constructed, respectively. The NS2 fragment containing deletion of NS2 103-262 amino acids was amplified by overlapping PCR with specific primers FNS2_HA_/RNS2d34, FNS2d34/RNS2_HA_ and then cloned into pKH3 using restriction enzyme *Hind*Ⅲ and *Sal*I digestion to generate pNS2_HA_/d34. The NS2 fragment containing deletion of NS2 263-457, or 361-457 amino acids were amplified by PCR with specific primers FNS2_HA_/RNS2dC262, or FNS2_HA_/RNS2dC360 and then cloned into pKH3 using *Hind*Ⅲ and *Sal*I digestion to generate pNS2_HA_/dC262, pNS2_HA_/dC360, respectively. All constructs were sequenced to confirm their identities. The primers for amplifying the fragments were shown in Supplementary Table 2.

**Supplementary Table 2** Primers for construction of p7 or NS2 mutants

| Primers | sequence (5'-3') | Constructs |
| --- | --- | --- |
| FP7dN1 | AGCTCTTAAGGCCACCATGATGAGGGATGAGCCTAT (*Afl*II) 113 | pP7_Myc_/dN1  (deleted p7 1-32 aa) |
| RP7_Myc_ | ATGCACGCGTACCCTTGGCAACCCCGCTAAC (*Mlu*I) |
| FP7dN2 | AGCTCTTAAGGCCACCATGACAGACATCGAGGTCGTAG (*Afl*II) 70 | pP7_Myc_/dN2  (deleted p7 1-17 aa) |
| RP7_Myc_ | ATGCACGCGTACCCTTGGCAACCCCGCTAAC (*Mlu*I) |
| FP7_Myc_ | AGCTCTTAAGGCCACCATGCTACCATTGGGCCAG (*Afl*II) | pP7_Myc_/dC1  (deleted p7 65-70 aa) |
| RP7dC1 | ATGCACGCGTCATAAGCAATGCCACTG (*Mlu*I) 189 |
| FP7_Myc_ | AGCTCTTAAGGCCACCATGCTACCATTGGGCCAG (*Afl*II) | pP7_Myc_/dC2  (deleted p7 41-70 aa) |
| RP7dC2 | TGCACGCGTTTTCTTTATAGGCTCATC (*Mlu*I) 120 |
| FP7_Myc_ | AGCTCTTAAGGCCACCATGCTACCATTGGGCCAG (*Afl*II) | pP7_Myc_/d1  (deleted p7 18-32 aa) |
| FP7d1A | CATGCTACCATTGGGCCAGGGTGAGGTAGTGTTGATAGGGAACTTAAT 44 |
| FP7d1B | TGTTGATAGGGAACTTAATCACCCACATGAGGGATGAGCCTATAAAG 117 |
| RP7_Myc_ | ATGCACGCGTACCCTTGGCAACCCCGCTAAC (*Mlu*I) |
| FpEF896 | ATTGACTAGTTATTAATAG (*Spel*I) | pNS2_HA_/d12  (deleted NS2 10-90 aa) |
| RNS2d12 | CTCCGCTGCCAACCGCCATCT 26 |
| FNS2d12 | TAGATGGCGGTTGGCAGCGGAGCACAGTGACAGGTAT 287 |
| RNS2_HA_ | CTAGAGTCGACTCTAAGCACCCAGCCAAG (*Sal*I) |
| FNS2_HA_ | TTCTAAGCTTGCCACCATGGGGGGAAAGATAGATGGC (*Hind*Ⅲ) | pNS2_HA_/d23  (deleted NS2 50-170 aa) |
| RNS2d23 | AGCTTTGTTAACTCGTAAGTTCTCAGGGTTGCCAC 530 |
| FNS2d23 | GAACTTACGAGTTAACAAAGC 529 |
| RNS2_HA_ | CTAGAGTCGACTCTAAGCACCCAGCCAAG (*Sal*I) |
| FNS2_HA_ | TTCTAAGCTTGCCACCATGGGGGGAAAGATAGATGGC (*Hind*Ⅲ) | pNS2_HA_/d34  (deleted NS2 103-262 aa) |
| RNS2d34 | CTTCAGTACCCTTATTAAAAAG 309 |
| FNS2d34 | CTTTTTAATAAGGGTACTGAAGAAGATCATAGATG 802 |
| RNS2_HA_ | CTAGAGTCGACTCTAAGCACCCAGCCAAG (*Sal*I) |
| FNS2_HA_ | TTCTAAGCTTGCCACCATGGGGGGAAAGATAGATGGC (*Hind*Ⅲ) | pNS2_HA_/dC262  (deleted NS2 263-457 aa) |
| RNS2dC262 | TAGAGTCGACGTGGAGGTAGTAAGACACT (*Sal*I) 786 |
| FNS2_HA_ | TTCTAAGCTTGCCACCATGGGGGGAAAGATAGATGGC (*Hind*Ⅲ) | pNS2_HA_/dC360  (deleted NS2 361-457 aa) |
| RNS2dC360 | TAGAGTCGACGTCTTCACAGACGGTG (*Sal*I) 1080 |
| FpEF896 | ATTGACTAGTTATTAATAG (*Spel*I) | pNS2_HA_/d1  (deleted NS2 10-40 aa) |
| RNS2d1 | GGTTGCCACCGTTATAACCAACCGCTGCCAACCGCCATCTAT 141 |
| FNS2d1 | TTGGTTATAACGGTGGCAACCC 142 |
| RNS2_HA_ | CTAGAGTCGACTCTAAGCACCCAGCCAAG (*Sal*I) |

The underlined sequences marked restriction enzyme sites

To investigate the effect of multiple amino acid mutation on protein-protein interaction, the p7 fragment containing TDI18/19/20AAA mutations was amplified by PCR using pSM/p7^TDI18/19/20AAA^ as a template with primers Fp7_Myc_ and Rp7_Myc_ (Supplementary Table 2 & Table 4). The amplified fragment was digested with restriction enzyme *Afl*II and *Mlu*I and cloned into pEF to generate pP7^TDI18/19/20AAA^. Similarly, the mutated eukaryotic expression plasmids pP7^EVV21/22/23AAA^ and pP7^YFY25/26/30AAA^ and pP7^V9A^ were contructed, respectively.

**Construction of full-length cDNA clones**

To construct the bicistronic genome cDNA clone (pSM/E2/IRES), the internal ribozyme entry site (IRES) sequence was amplified by PCR using pHAGE-IZsGreen as a template with primers F-IRES and R-IRES. The amplified IRES fragment was fused to the site between E2 and p7 by overlapping PCR with primers FE2/2409 and RE2/3541, FP7/3578 and FNS2/6426. The chemeric fragment containing IRES replaced the restriction fragment *Spel*I^2419^-*BamH*I^6436^ of pSM to generate pSM/E2/IRES. The *Spel*I^2419^-*Kpn*I^4448^ fragment containing deletion of p7 15-51 amino acids was amplified by overlapping PCR with primers FE2/2409 and RP7/3581, FP7/3723 and RNS2/4440. After digestion with restriction enzyme *Spel*I and *Kpn*I, the fragment substituted the *Spel*I^2419^-*Kpn*I^4448^ fragment of pSM to generate pSM/Δp7^15-51^. The fragment containing E2 ASG to NSR mutations was amplified by overlapping PCR with primers FE2/2409 and RE2/3547, FP7/3569 and RNS2/4440. After digestion with restriction enzyme *Spel*I and *Kpn*I, the fragment substituted the *Spel*I^2419^-*Kpn*I^4448^ of pSM to generate pSM/E2^ASG/NSR^. The primers for construction of pSM/E2/IRES, pSM/Δp7^15-51^, pSM/E2^ASG/NSR^ were shown in Supplementary Table 3.

**Supplementary Table 3** Primers for construction of pSM/E2/IRES, pSM/Δp7^15-51^, pSM/E2^ASG/NSR^ (A371N, G373R)

| Primers | sequence (5'-3') | Constructs |
| --- | --- | --- |
| FE2/2409 | TATGGCTGCTACTAGTAACTGG (*Spel*I) | pSM/E2/IRES  (inserted IRES between E2 and p7) |
| RE2/3541 | AGGGGGGGGGGAGGGAGAGGCTAACCAGCGGCGAGTTGTTCT |
| F-IRES | CCTCTCCCTCCCCCCCCCCTAACG |
| R-IRES | TGTGGCCATATTATCATCGT |
| FP7/3578 | CACGATGATAATATGGCCACAATGTTACCATTGGGCCAGGGT |
| RNS2/6426 | CGGTGGATCCTCTCCACTAT (*BamH*I) |
| FE2/2409 | TATGGCTGCTACTAGTAACTGG (*Spel*I) | pSM/Δp7^15-51^  (deleted p7 15-51 aa) |
| Rp7/3581 | TGACTGGATTTAAGTTCCCTATCAACACTAC |
| Fp7/3723 | AGGGAACTTAAATCCAGTCAAGACTATAAC |
| RNS2/4440 | GGCAACATGGTACCTGTTATTG (*Kpn*I) |
| FE2/2409 | TATGGCTGCTACTAGTAACTGG (*Spel*I) | pSM/E2^ASG/NSR^  (E2/A371N, G373R) |
| RE2/3547 | TCACCCTGGCCCAATGGTAGACGAGCGTTGAGT |
| Fp7/3569 | CTACCATTGGGCCAGGGTG |
| RNS2/4440 | GGCAACATGGTACCTGTTATTG (*Kpn*I) |

The underlined sequences marked restriction enzyme sites.

Mutations in p7 N-terminus were introduced by overlapping PCR using standard procedures and engineered into monocistronic construct (pSM), respectively. Briefly, the fragment *Spel*I^2419^-*Kpn*I^4448^ containing p7 L1A site-directed mutagenesis was amplified by overlapping PCR using pSM as a template with primers FE2/2409 and RE2/3536, FP7^L1A^ and RNS2/4440. The amplified fragment was digested with *Spel*I/*Kpn*I and then substituted the fragment *Spel*I^2419^-*Kpn*I^4448^ of pSM to generate pSM/p7^L1A^. Other pSM/p7 mutants were constructed using the similar strategy with site-directed mutagenesis primers. Based on bicistronic cDNA clone pSM/E2/IRES, we constructed the corresponding p7 mutants using the same strategy. All constructs were sequenced to confirm their identities. The primers for amplifying the fragments were shown in Supplmentary Table 4.

**Supplementary Table 4** Primers for construction of infectious cDNA clones with p7 mutation

| Primers | sequence (5'-3') | Constructs |
| --- | --- | --- |
| FE2/2409 | TATGGCTGCTACTAGTAACTGG (*Spel*I) | pSM/p7^L1A^  pSM/p7^P2A^  pSM/p7^L3A^  pSM/p7^G4A^ |
| RE2/3536 | ACCAGCGGCGAGTTGTTCTGTTAG |
| Fp7^L1A^  Fp7^P2A^  Fp7^L3A^  Fp7^G4A^ | AACAGAACAACTCGCCGCTGGT***GC****A*CCATTGGGCCAGG (CTA→***G****CA*)  AACAGAACAACTCGCCGCTGGTCTA***G****CA*TTGGGCCAGG (CCA→***G****CA*)  AACAGAACAACTCGCCGCTGGTCTACCA***GC****G*GGCCAGG (TTG→***G****CG*)  AACAGAACAACTCGCCGCTGGTCTACCATTG*G****C****C*CAGG (GGC→*G****C****C*) |
| RNS2/4440 | GGCAACATGGTACCTGTTATTG (*Kpn*I) |
| FE2/2409 | TATGGCTGCTACTAGTAACTGG (*Spel*I) | pSM/p7^Q5A^  pSM/p7^G6A^  pSM/p7^E7A^  pSM/p7^V8A^  pSM/p7^V9A^ |
| RE2/3552 | GCCCAATGGTAGACCAGCGG |
| Fp7^Q5A^  Fp7^G6A^  Fp7^E7A^  Fp7^V8A^  Fp7^V9A^ | CCGCTGGTCTACCATTGGGC***GC****G*GGTGAGGTAGTGTTGAT (CAG→***GC****G*)  CCGCTGGTCTACCATTGGGCCAG*G****C****T*GAGGTAGTGTTGAT (GGT→*G****C****T*)  CCGCTGGTCTACCATTGGGCCAGGGT*G****C****G*GTAGTGTTGAT (GAG→*G****C****G*)  CCGCTGGTCTACCATTGGGCCAGGGTGAG*G****C****A*GTGTTGAT (GTA→*G****C****A*)  CCGCTGGTCTACCATTGGGCCAGGGTGAGGTA*G****C****G*TTGAT (GTG→*G****C****G*) |
| RNS2/4440 | GGCAACATGGTACCTGTTATTG (*Kpn*I) |
| FE2/2409 | TATGGCTGCTACTAGTAACTGG (*Spel*I) | pSM/p7^T18A^  pSM/p7^D19A^  pSM/p7^I20A^  pSM/p7^E21A^  pSM/p7^V22A^  pSM/p7^V23A^ |
| Rp7/3590 | GTGGGTGATTAAGTTCCCTAT |
| Fp7^T18A^  Fp7^D19A^  Fp7^I20A^  Fp7^E21A^  Fp7^V22A^  Fp7^V23A^ | ATAGGGAACTTAATCACCCAC***G****CA*GACATCGAGG (ACA→***G****CA*)  ATAGGGAACTTAATCACCCACACA*G****C****C*ATCGAGG (GAC→*G****C****C*)  ATAGGGAACTTAATCACCCACACAGAC***GC****C*GAGG (ATC→***GC****C*)  ATAGGGAACTTAATCACCCACACAGACATC*G****C****G*G (GAG→*G****C****G*)  ATAGGGAACTTAATCACCCACACAGACATCGAG*G****C****C*GTAGTAT (GTC→*G****C****C*)  ATAGGGAACTTAATCACCCACACAGACATCGAGGTC*G****C****A*GTAT (GTA→*G****C****A*) |
| RNS2/4440 | GGCAACATGGTACCTGTTATTG (*Kpn*I) |
| FE2/2409 | TATGGCTGCTACTAGTAACTGG (*Spel*I) | pSM/p7^Y25A^  pSM/p7^F26A^  pSM/p7^Y30A^ |
| Rp7/3610 | TACTACGACCTCGATGTCTGTG |
| Fp7^Y25A^  Fp7^F26A^  Fp7^Y30A^ | CACAGACATCGAGGTCGTAGTA***GC****T*TTCTTACTACTCTATT (TAT→***GC****T*)  CACAGACATCGAGGTCGTAGTATAT***GC****C*TTACTACTCTATT (TTC→***GC****C*)  GACATCGAGGTCGTAGTATATTTCTTACTACTC***GC****T*TTGGTCATGAGG (TAT→***GC****T*) |
| RNS2/4440 | GGCAACATGGTACCTGTTATTG (*Kpn*I) |
| FE2/2409 | TATGGCTGCTACTAGTAACTGG (*Spel*I) | pSM/p7^TDI18/19/20AAA^  pSM/p7^EVV21/22/23AAA^ |
| Rp7/3590 | GTGGGTGATTAAGTTCCCTAT |
| Fp7^TDI18/19/20AAA^  Fp7^EVV21/22/23AAA^ | ATAGGGAACTTAATCACCCAC***G****CAG****C****C****GC****C*GAGGTCG (ACA→***G****CA*, GAC→***GC****C*, ATC→***GC****C*)  ATAGGGAACTTAATCACCCACACAGACATC*G****C****GG****C****CG****C****A*GTAT (GAG→*G****C****G*, GTC→*G****C****C*, GTA→*G****C****A*) |
| RNS2/4440 | GGCAACATGGTACCTGTTATTG (*Kpn*I) |
| FE2/2409 | TATGGCTGCTACTAGTAACTGG (*Spel*I) | pSM/p7^YFY25/26/30AAA^ |
| Rp7/3610 | TACTACGACCTCGATGTCTGTG |
| Fp7^YFY25/26/30AAA^ | CACAGACATCGAGGTCGTAGTA***GC****T****G****CC*TTACTACTC***GC****T*TTGGT (TAT→***GC****T*, TCC→***G****CC*, TAT→***GC****T*) |
| RNS2/4440 | GGCAACATGGTACCTGTTATTG (*Kpn*I) |
| FE2/2409 | TATGGCTGCTACTAGTAACTGG (*Spel*I) | pSM/E2/IRES/p7^L1A^  pSM/E2/IRES/p7^P2A^  pSM/E2/IRES/p7^L3A^  pSM/E2/IRES/p7^G4A^ |
| R-IRES | TGTGGCCATATTATCATCGT |
| FE2/IRES/p7^L1A^  FE2/IRES/p7^P2A^  FE2/IRES/p7^L3A^  FE2/IRES/p7^G4A^ | ACGATGATAATATGGCCACAATG***GC****A*CCATTGGGCCAGG (CTA→***GC****A*)  ACGATGATAATATGGCCACAATGCTA***G****CA*TTGGGCCAGG (CCA→***G****CA*)  ACGATGATAATATGGCCACAATGCTACCA***GC****G*GGCCAGG (TTG→***GC****G*)  ACGATGATAATATGGCCACAATGCTACCATTG*G****C****C*CAGG (GGC→*G****C****C*) |
| RNS2/6426 | CGGTGGATCCTCTCCACTAT (*BamH*I) |
| FE2/2409 | TATGGCTGCTACTAGTAACTGG (*Spel*I) | pSM/E2/IRES/p7^Q5A^  pSM/E2/IRES/p7^G6A^  pSM/E2/IRES/p7^E7A^  pSM/E2/IRES/p7^V8A^  pSM/E2/IRES/p7^V9A^ |
| Rp7/3571 | GCCCAATGGTAGCATTGTGG |
| FE2/IRES/p7^Q5A^  FE2/IRES/p7^G6A^  FE2/IRES/p7^E7A^  FE2/IRES/p7^V8A^  FE2/IRES/p7^V9A^ | CCACAATGCTACCATTGGGC***GC****G*GGTGAGGTAGTGTTGAT (CAG→***GC****G*)  CCACAATGCTACCATTGGGCCAG*G****C****T*GAGGTAGTGTTGAT (GGT→*G****C****T*)  CCACAATGCTACCATTGGGCCAGGGT*G****C****G*GTAGTGTTGAT (GAG→*G****C****G*)  CCACAATGCTACCATTGGGCCAGGGTGAG*G****C****A*GTGTTGAT (GTA→*G****C****A*)  CCACAATGCTACCATTGGGCCAGGGTGAGGTA*G****C****G*TTGAT (GTG→*G****C****G*) |
| RNS2/6426 | CGGTGGATCCTCTCCACTAT (*BamH*I) |

The underlined sequences marked restriction enzyme sites. The changed nucleotides showed in parentheses.


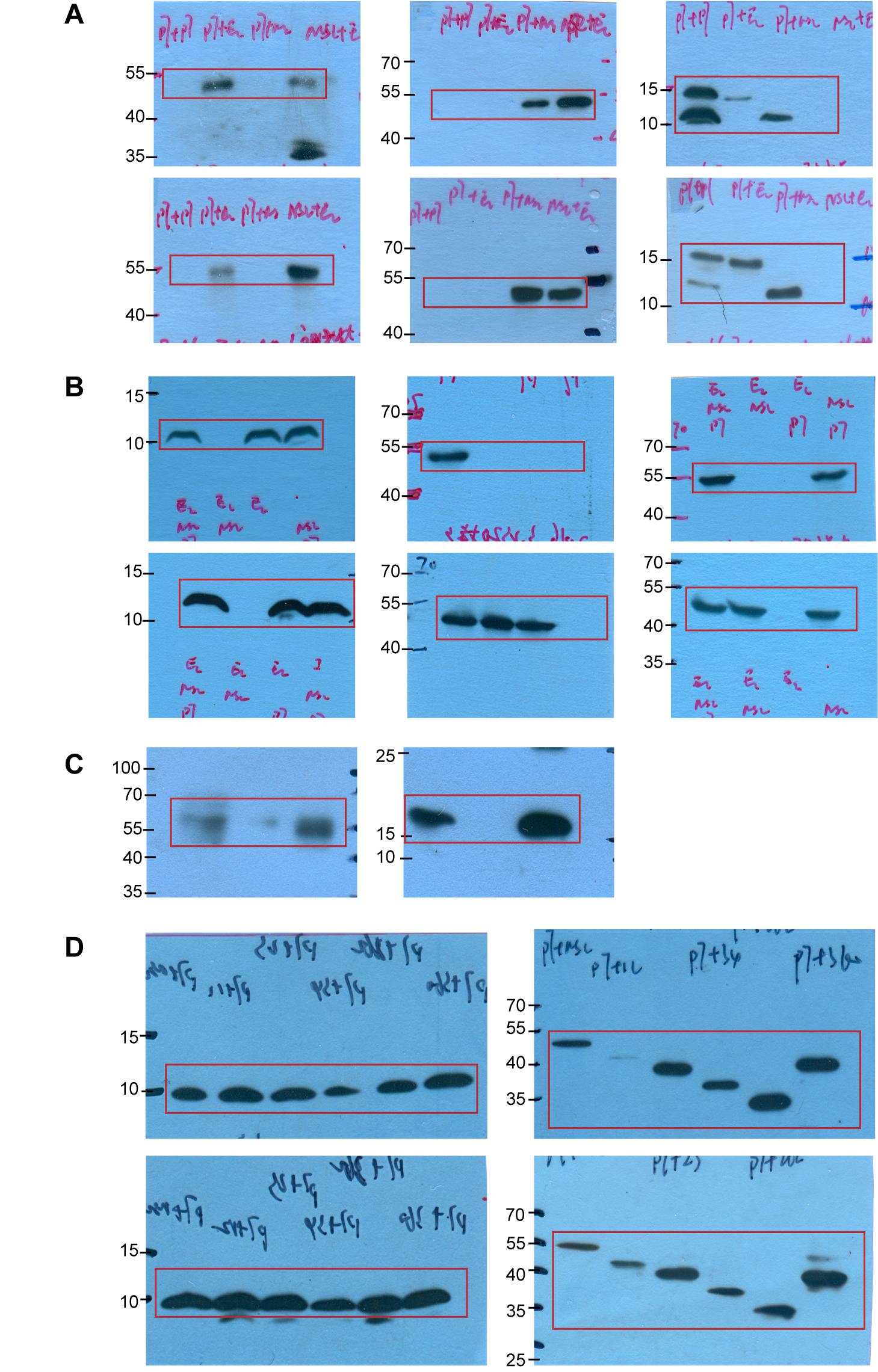

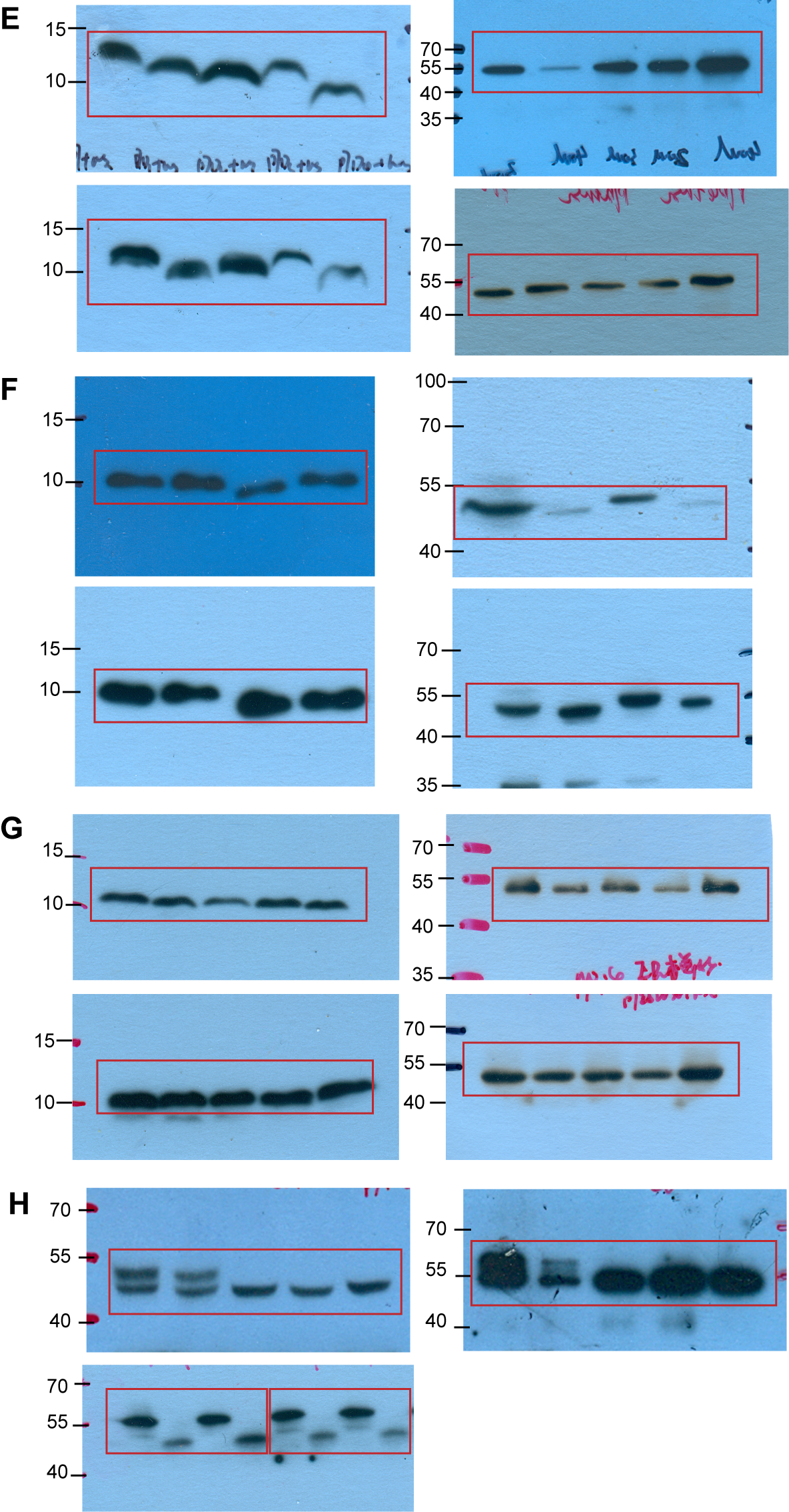


**Supplementary Fig.1.** Original scans of western blotting results. The boxed regions indicate the bands shown in the figures. (A) Fig. 1A; (B) Fig. 1B; (C) Fig. 2C; (D) Fig. 2B; (E) Fig. 2C; (F) Fig. 3D; (G) Fig. 4D; (H) Fig. 5A.


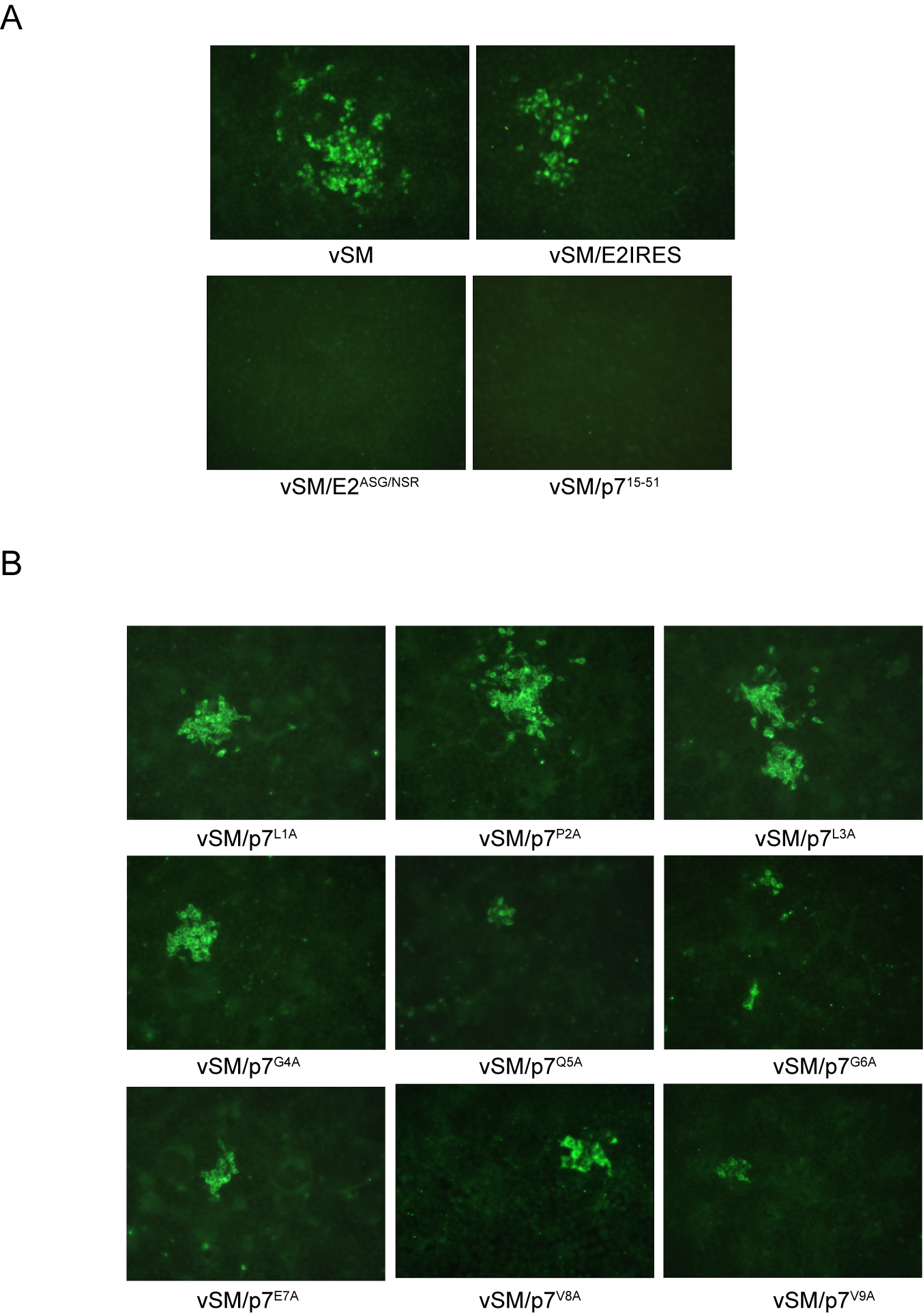

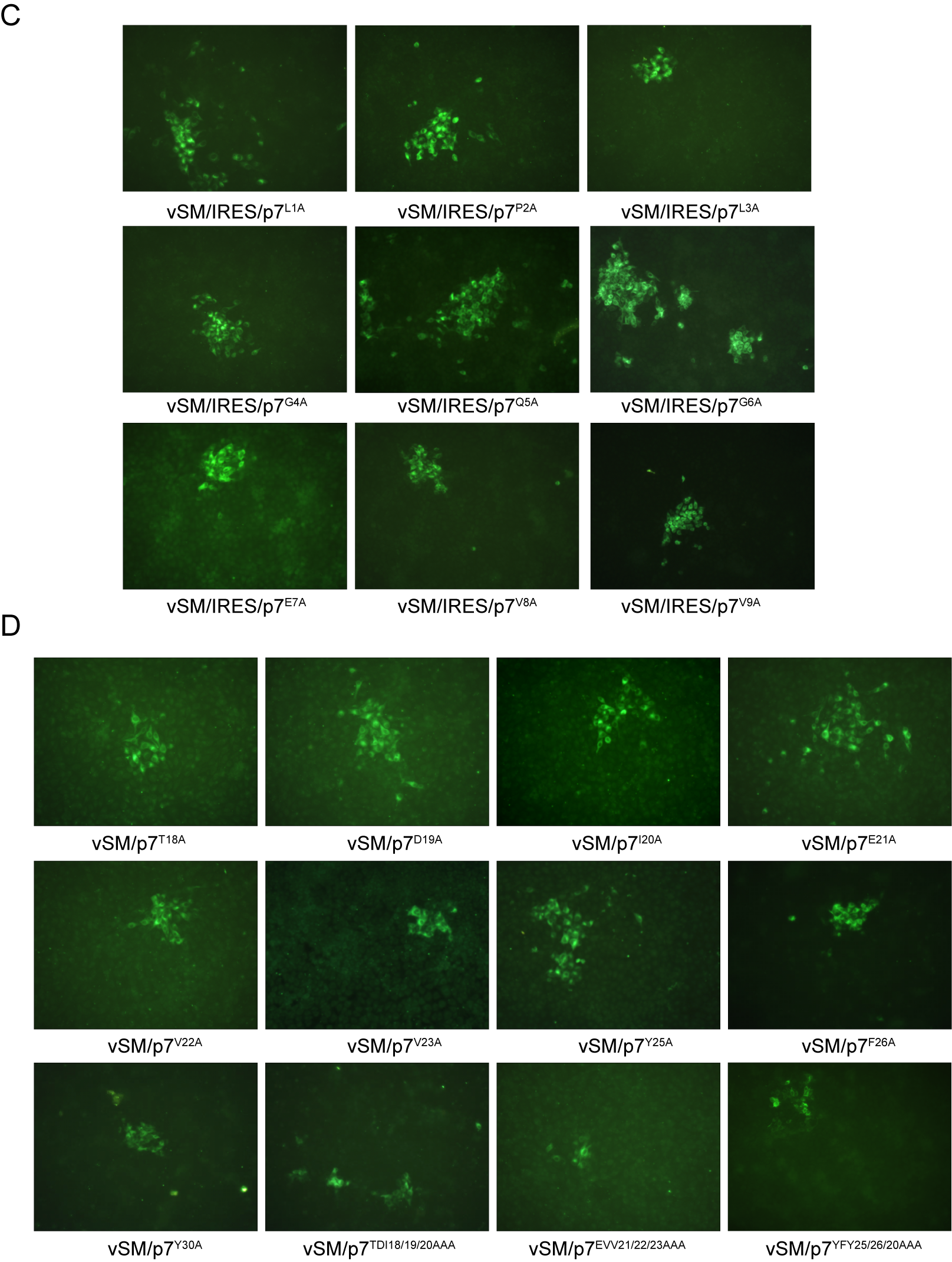


**Supplementary Fig.2.** Identification of infectious CSFVs from the PK15 cells transfected with wt and mutated p7 transcripts. At 72 hpt, the transfected cells were fixed and analyzed by IF staining using NS3-specific antibody.

**Supplementary Fig. 3.** A proposed model for a complex of E2, p7 and NS2 associated with ER membranes. The protein p7, E2 and NS2 of CSFV are co-located in the plasma membrane of ER and form a protein-protein complex. The gray double-headed arrows indicate the direct interactions. Three mutants containing TDI18/19/20AAA, EVV21/22/23AAA, and YFY25/26/30AAA (marked) located in the TM1 domain of p7 interacts with the TM1 domain of NS2; 1-9 amino acid residues of p7 N-terminus mediate the E2p7 processing.
